# Influence of Lumbar Muscle Fatigue on Trunk Adaptations during Sudden External Perturbations

**DOI:** 10.3389/fnhum.2016.00576

**Published:** 2016-11-14

**Authors:** Jacques Abboud, François Nougarou, Arnaud Lardon, Claude Dugas, Martin Descarreaux

**Affiliations:** ^1^Département d’Anatomie, Université du Québec à Trois-RivièresTrois-Rivières, QC, Canada; ^2^Département de Génie Électrique, Université du Québec à Trois-RivièresTrois-Rivières, QC, Canada; ^3^Institut Franco-Européen de ChiropraxieIvry-Sur-Seine, France; ^4^Département des Sciences de l’Activité Physique, Université du Québec à Trois-RivièresTrois-Rivières, QC, Canada

**Keywords:** high-density electromyography, spinal stability, muscle fatigue, reflex, habituation

## Abstract

**Introduction**: When the spine is subjected to perturbations, neuromuscular responses such as reflex muscle contractions contribute to the overall balance control and spinal stabilization mechanisms. These responses are influenced by muscle fatigue, which has been shown to trigger changes in muscle recruitment patterns. Neuromuscular adaptations, e.g., attenuation of reflex activation and/or postural oscillations following repeated unexpected external perturbations, have also been described. However, the characterization of these adaptations still remains unclear. Using high-density electromyography (EMG) may help understand how the nervous system chooses to deal with an unknown perturbation in different physiological and/or mechanical perturbation environments.

**Aim**: To characterize trunk neuromuscular adaptations following repeated sudden external perturbations after a back muscle fatigue task using high-density EMG.

**Methods**: Twenty-five healthy participants experienced a series of 15 sudden external perturbations before and after back muscle fatigue. Erector spinae muscle activity was recorded using high-density EMG. Trunk kinematics during perturbation trials were collected using a 3-D motion analysis system. A two-way repeated measure ANOVA was conducted to assess: (1) the adaptation effect across trials; (2) the fatigue effect; and (3) the interaction effect (fatigue × adaptation) for the baseline activity, the reflex latency, the reflex peak and trunk kinematic variables (flexion angle, velocity and time to peak velocity). Muscle activity spatial distribution before and following the fatigue task was also compared using *t*-tests for dependent samples.

**Results**: An attenuation of muscle reflex peak was observed across perturbation trials before the fatigue task, but not after. The spatial distribution of muscle activity was significantly higher before the fatigue task compared to post-fatigue trials. Baseline activity showed a trend to higher values after muscle fatigue, as well as reduction through perturbation trials. Main effects of fatigue and adaptation were found for time to peak velocity. No adaptation nor fatigue effect were identified for reflex latency, flexion angle or trunk velocity.

**Conclusion**: The results show that muscle fatigue leads to reduced spatial distribution of back muscle activity and suggest a limited ability to use across-trial redundancy to adapt EMG reflex peak and optimize spinal stabilization using retroactive control.

## Introduction

Over the past years, several studies have shown that neuromuscular adaptations are observed under the influence of back muscle fatigue (Allison and Henry, 2001; Boyas and Guével, 2011; Monjo et al., 2015). Indeed, some authors have reported that a reorganization of motor strategies is used to prevent the onset of muscle fatigue (Fuller et al., 2011) and that such adaptations in muscle activity recruitment patterns are present, such as co-contraction phenomena (Allison and Henry, 2001), or within muscle changes in recruitment patterns, suggesting a spatial dependency in the control of motor units in the erector spinae (Tucker et al., 2009; Abboud et al., 2014). These neuromuscular adaptations have been also reported when participants are asked to perform a voluntary perturbation, such as goal-directed movements. Previous studies have shown that, in the presence of muscle fatigue, compensatory neuromuscular adaptations occur in order to maintain the task requirement (Côté et al., 2002; Missenard et al., 2009). Such neuromuscular strategies are part of the feedforward control, which allows the central nervous system to predict the muscle activation needed to achieve a desired motor task (Shadmehr and Mussa-Ivaldi, 1994). On the other hand, when subjected to unpredictable perturbations, neither feedback, nor anticipation strategies are sufficient to adjust movement on-line. Determining the influence of muscle fatigue during an unpredictable perturbation is therefore of great interest.

More specifically, this study focusses on understanding neuromuscular adaptations to unexpected trunk perturbation, which are believed to affect spinal stability. In everyday life, the human body is constantly under the influence of mechanical forces applied in different directions, sometimes unexpected and continuously triggering postural adjustments. Examples of such spinal perturbations can be drawn from various common activities such as sport contacts, tripping, slipping, weight lifting, etc. Panjabi (1992) described spinal stability as a complex mechanism involving three essential components: spinal muscles, passive spinal tissues and neuromuscular control (Panjabi, 1992). Alterations, such as physiological and/or mechanical ones, of one or more of these components have been shown to be a direct or indirect manifestation of spinal instability.

Despite the number of studies that have investigated fatigue and unexpected loading effects on spinal stability, results vary from one study to the other (Granata et al., 2001, 2004; Chow et al., 2004; Herrmann et al., 2006; Mawston et al., 2007; Grondin and Potvin, 2009; Dupeyron et al., 2010; Sánchez-Zuriaga et al., 2010). Such differences could be partly due to methodological choices in trunk perturbation experimental protocols, such as participant positions (standing vs. sitting), familiarization of the external perturbation, perturbation magnitudes, etc. Furthermore, variables selected to assess neuromuscular responses to a sudden trunk perturbation are far from consistent across studies. The most common variables used to assess the effect of unexpected trunk loading under muscle fatigue are baseline muscle activity, reflex latency and reflex amplitude. In a context of unexpected trunk perturbation, current evidence shows inconsistencies in baseline activity responses under the influence of muscle fatigue (Granata et al., 2001, 2004; Herrmann et al., 2006; Mawston et al., 2007; Grondin and Potvin, 2009; Dupeyron et al., 2010) with studies showing no adaptation after a back fatigue task (Herrmann et al., 2006; Mawston et al., 2007; Dupeyron et al., 2010), while other ones reveal an increase in baseline activity with muscle fatigue (Granata et al., 2001, 2004; Grondin and Potvin, 2009). On the other hand, most studies investigating neuromuscular responses following an unexpected trunk perturbation showed that reflex latency is not affected by the presence of low back muscle fatigue (Granata et al., 2004; Herrmann et al., 2006; Dupeyron et al., 2010; Sánchez-Zuriaga et al., 2010). As for the reflex amplitude of low back muscles, it was found not to be affected by muscle fatigue in several studies (Granata et al., 2004; Grondin and Potvin, 2009; Sánchez-Zuriaga et al., 2010), whereas few studies found a higher back reflex amplitude following a fatigue protocol (Herrmann et al., 2006; Dupeyron et al., 2010). Overall, the effect of muscle fatigue on neuromuscular adaptations during unexpected loading remains unclear.

Inconsistencies reported in the literature regarding neuromuscular responses under muscle fatigue during unexpected perturbation could also be explained by the fact that most of the studies have been limited by the amplitude and frequency behavior because of the use of classic bipolar electromyography (EMG), which covers only a small portion of the explored muscle. Recent technologies, such as high-density surface EMG (sEMG), because it can cover a large surface area, offer a unique perspective on muscle activity spatial distribution (Zwarts and Stegeman, 2003; Merletti et al., 2008; Holobar et al., 2009; Martinez-Valdes et al., 2016). Indeed, data extracted from high-density sEMG have enabled the mapping of muscle activity recruitment distribution in the low back region during a voluntary contraction. Results have revealed a shift in muscle activity spatial distribution to the lateral-caudal direction in the low back region during muscle fatigue (Tucker et al., 2009). This migration in muscle activity distribution could be associated with changes in muscle fiber recruitment to avoid overloading of the same fibers (Rantanen et al., 1994). Adopting non-uniform muscle activity recruitment may help participants develop motor strategies and facilitate adaptation to different physiological and/or mechanical perturbations of the spine. Low back muscle activity measured by high-density sEMG has also been shown to discriminate patients with chronic low back pain from healthy individuals through different motor tasks (Abboud et al., 2014; Falla et al., 2014). To our knowledge, no study has investigated muscle activity reflex variables with high-density sEMG.

Reflex muscle activity has also been studied following a series of unexpected external perturbations. The first research exploring this question was conducted by Nashner (1976), who showed that neuromuscular adaptations, such as the attenuation of lower limb muscle reflex activation, occur following repeated ankle dorsiflexion to improve postural balance. More recently, similar results have been reported in presence of several unexpected external perturbations of the cervical region (Blouin et al., 2003; Siegmund et al., 2003). A reduction of neck muscle activity was observed across perturbation trials (Blouin et al., 2003; Siegmund et al., 2003). Back muscles seem to follow a similar response pattern during unexpected forward perturbation. Skotte et al. (2004) showed a reduction of the average erector spinae EMG amplitude from the first trial to the next one. A more recent study reported similar results in paraspinal muscle responses to a series of unexpected tilts from a surface platform (Oude Nijhuis et al., 2010). The authors observed that EMG amplitude responses adapted rapidly between the first two trials, whereas adaptation was more gradual over the next trials (Oude Nijhuis et al., 2010). Based on these results, it could be suggested that, whenever possible, the central nervous system attempts to minimize unnecessary or excessive responses to perturbation.

Moreover, adaptations throughout repetitions of the same perturbation have been previously shown to compensate for the effect of muscle fatigue (Takahashi et al., 2006; Kennedy et al., 2012). Such compensations were indeed observed following upper limb and ankle muscle fatigue when movement accuracy and postural stability were respectively maintained (Takahashi et al., 2006; Kennedy et al., 2012). However, in the study of Kennedy et al. (2012), the authors did not assess adaptation across repeated perturbation trials. Participants had to perform few perturbation trials to allow habituation to perturbations, to limit changes caused by habituation to the motor task. On the other hand, Takahashi et al. (2006) studied adaptation across perturbation trials. The unexpected perturbation was applied while participants reached to a target and results showed that even when participants were in a state of fatigue, they were still able to reach the target and adapt to perturbations.

The assessment of neuromuscular adaptations when the patients are submitted to the same perturbation during a rehabilitation protocols could be used to monitor progress and adaptations. As suggested by Hodges and Tucker (2011), adaptations to pain have immediate and short term benefit for the spinal system. They may, however, have detrimental long-term consequences and should therefore be monitored and perhaps treated in rehabilitation. The aim of the present study was to characterize trunk neuromuscular adaptations in response to a sudden external perturbation after a back fatigue task. Its second aim was to identify if trunk neuromuscular control can be modulated by a previous instability experience in the presence of back muscle fatigue using high-density sEMG. Based on current evidence, it was hypothesized that back muscle fatigue would alter trial-to-trial neuromuscular adaptions during a series of repeated sudden external perturbations. Moreover, it was hypothesized that trial-to-trial neuromuscular adaptations would lead to a migration of muscle activity within the erector spinae, and that this spatial muscle activity migration would be limited in the presence of muscle fatigue.

## Materials and Methods

### Recruitment

Twenty-five healthy adult participants (22 men and 3 women) were recruited from the university community. Participants with one of the following criteria were excluded: history of acute/chronic thoracic or low back pain in the past 6 months, ankylosing spondylitis, trunk neuromuscular disease, inflammatory arthritis, scoliosis (≥15°), and previous spinal surgery. Participant mean (*M*) age, height, weight and BMI were respectively 26.8 (standard deviation (*SD*) = 5.5) years, *M* = 1.76 (*SD* = 0.7) m, *M* = 76.6 (*SD* = 12.1) kg and *M* = 24.5 (*SD* = 3.1) kg/m^2^. The project received approval from the university’s ethics committee for research with humans (Comité d’éthique de la recherche avec des êtres humains). All participants gave their written informed consent prior to their participation in this study.

### Experimental Protocol

**Figure 1:**
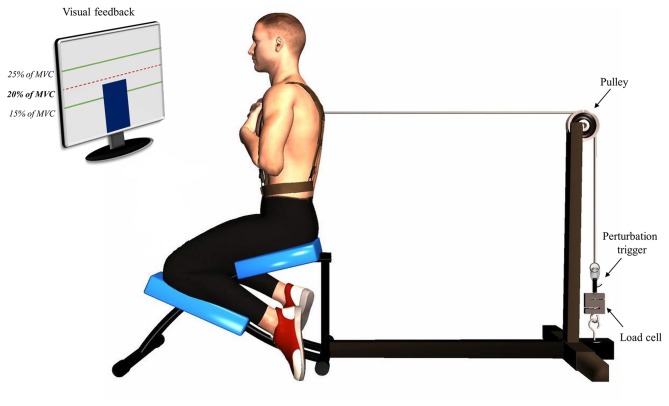
**Illustration of the perturbation protocol.** Participants were positioned in a semi-seated position with their trunk attached to a manual trigger by a cable using a pulley system. A visual feedback was provided using a screen indicating the target of 20% of their trunk flexion maximal voluntary contraction.

First, EMG electrodes and light-emitting diodes from the 3-D motion analysis system were installed over the participants. The experimentation protocol was divided in three phases: pre-fatigue perturbations, fatigue protocol and post-fatigue perturbations. Before the first phase, two or three isometric trunk flexion maximal voluntary contractions (MVC) were performed, as well as two or three isometric maximal contractions in trunk extension. The third trial of MVC was only performed if the participants’ second MVC was superior to the first one. MVC was assessed in a semi-seated position on a modified ergonomic back chair custom-built for the study (Figure 1). For the trunk flexion MVC, participants were asked to pull anteriorly against a cable. Their trunk was attached at T8 level to a load cell (Model LSB350; Futek Advanced Sensor Technology Inc., Irvine, CA, USA) with a cable using a pulley system. As for trunk extension MVC, participants were asked to pull posteriorly against the cable. Since no warm up exercise was provided, participants were invited to perform some trunk extension and flexion contractions before the MVC protocol. The goal of these contractions was to help participants familiarize with the MVC protocol.

The first phase of the protocol, pre-fatigue perturbations, consisted in a series of 15 sudden external perturbations. Participants were asked to adopt the same position as the one used during the MVC protocol (Figure 1). Their trunk was attached to a perturbation trigger with a cable using a pulley system. This set-up was designed to generate a posterior to anterior perturbation of the trunk. The trigger was connected to a load cell (Model LSB350; Futek Advanced Sensor Technology Inc., Irvine, CA, USA) to objectively measure the pulling trunk flexion force exerted by participants. Participants were asked to maintain a pulling force corresponding to 20% of their trunk flexion MVC and to return to the neutral equilibrium position as quickly as possible after the perturbation. The perturbation magnitude corresponded on average to 55N, ranging from 37N to 76N across participants, which is similar to the perturbation magnitude used in similar perturbation protocols (Radebold et al., 2000; Reeves et al., 2005). The higher value of trunk flexion MVC was used to determine the target force for the perturbation protocol. To help participants reach and stabilize their pulling target force, a visual feedback was provided using a screen indicating real time traction (Figure 1). Once the force was stabilized, one of the assessors triggered the perturbation after 1, 3 or 5 s according to a random sequence. The perturbation sequences were different for each participant and each condition (pre- and post-fatigue) to avoid any anticipation of the perturbation onset.

Following the first phase, the fatigue task consisted of a modified version of the Sorensen endurance test (Champagne et al., 2009). Participants were asked to lay in a prone position on a 45° Roman chair, with the iliac crests aligned with the chair cushion edge. In order to quickly induce muscular fatigue, participants had to lift a 12.5-kg weight plate during the task, and hold it as close as possible to their chest. The participants’ trunk was maintained unsupported in a horizontal position relative to the ground for as long as possible. The investigators gave similar verbal encouragement to all subjects. Perceived effort scale (6–20; Borg, 1982), measuring the intensity of the fatigue task, was rated by each participant at the end of the fatigue test. Before and after the fatigue protocol, an MVC protocol was performed in the same position as the fatigue task; a belt was fixed to the ground and installed over the participants’ shoulders and they were asked to perform a maximal trunk extension contraction against the belt.

The last phase of the experimentation, post-fatigue perturbations, was performed immediately after the fatigue protocol. To avoid the attenuation of muscle fatigue effects, the transition between the fatigue protocol and the second series of perturbation was made as quickly as possible. The time needed between the end of the fatigue protocol and the acquisition of the data ranged from 2 min to 4 min. In this last phase, participants were submitted to 15 more perturbations, identical to the ones received before the fatigue protocol. This part of the experiment lasted no more than 8 min. The total duration of the last phase of the experimentation ranged from 10 min to 12 min.

### Data Acquisition

Two different EMG acquisition systems were used to record trunk muscle activity. sEMG of the right and left erector spinae muscles was recorded using two adhesive matrices of 64 electrodes (model ELSCH064; LISiN-OT Bioelettronica; Torino, Italy). The array grid consisted of 64 electrodes placed in an 8 × 8 matrix (10 mm inter-electrode distance). The center of each grid was located at L3 level, and one bracelet ground electrode was placed on the right wrist. The bipolar EMG signals were amplified (64-channel sEMG amplifier, SEA 64, LISiN-OT Bioelettronica; Torino, Italy; –3 dB bandwidths 10–500 Hz) by a factor of 5000 during the perturbations’ protocol, while a 2000 factor was applied during the fatigue protocol. The signal was sampled at 2048 Hz and converted to digital form by a 12-bit A/D converter. Rectus abdominis and external obliquus abdominis muscle activity were recorded using a differential Ag sEMG sensor with a common mode rejection ratio of 92 dB at 60 Hz, a noise level of 1.2 μV, a gain of 10 V/V ± 1%, a bandwidth of 20–450 ± 10% (Model DE2.1, Delsys Inc., Boston, MA, USA), amplified by a factor 10,000 and sampled at 2048 Hz with a 12-bit A/D converter (PCI 6024E, National Instruments, Austin, TX, USA). Each bipolar signal was digitally band-pass filtered in the frequency bandwidth-30-450 Hz (2nd order Butterworth filter). Notch filters were also applied to eliminate the 60 Hz and 100 Hz power line interference and its harmonics. To avoid inter-rater variability, anatomical structures palpation and placement of electrodes were assessed by the same investigator for all participants. The electrode position for rectus abdominis and external obliquus was located over the midsection of the muscle and parallel to the fibers orientation, as described by Criswell and Cram (2011). Before the application of an electrode, skin impedance was reduced by shaving body hair, gently exfoliating the skin with fine-grade sandpaper (Red DotTrace Prep, 3 M; St. Paul, MN, USA) and wiping the skin with alcohol swabs. The data from both EMG acquisition systems were collected using the OT Bioelettronica custom software and processed by Matlab (MathWorks; Natick, MA, USA). Trunk extensor and trunk flexor myoelectric signals from EMG were normalized with respect to the trunk extension and flexion MVC values.

Finally, trunk kinematics during perturbation trials was collected using a 3-D motion analysis system (Optotrak Certus, Northern Digital, Waterloo, ON, Canada). Light-emitting diodes were positioned on the left side of the participants over two anatomical landmarks: (1) L1, (2) T11. A third light-emitting diode was placed on the trigger perturbation. Data were sampled at 100 Hz and low-pass filtered by a dual-pass, fourth-order Butterworth filter with a cut-off frequency of 5 Hz. EMG data and kinematic data were synchronized through a signal triggered by OT Bioelettronica software and Matlab (MathWorks). Both EMG and kinematics were recorded for 10 s.

### Data Analysis

From high-density sEMG signals, in order to confirm the presence of erector spinae muscle fatigue, the mean normalized slope of the median frequency (MDF; mean of the 64 electrodes of each matrix) was calculated from adjacent non-overlapping signal epochs of 0.5 s. Moreover, the percentage of EMG amplitude root mean square (RMS) diminution between the MVC pre-fatigue and MVC post-fatigue was calculated. Finally, four variables were extracted: the baseline activity, the reflex latency, the reflex peak and the area of muscle activity spatial distribution. Left and right erector spinae muscles were analyzed separately. From abdominal EMG signals, reflex activity was also computed. For all variables, reflex responses latencies superior to 300-ms from the perturbation onset were not analyzed to avoid inclusion of any voluntary responses.

#### Baseline Activity

Erector spinae baseline activity was quantified as the mean EMG amplitude RMS using a 500-ms window prior to the onset of the perturbation. The mean of all electrodes for each high-density sEMG (left and right) was calculated.

#### Reflex Latency

**Figure 2:**
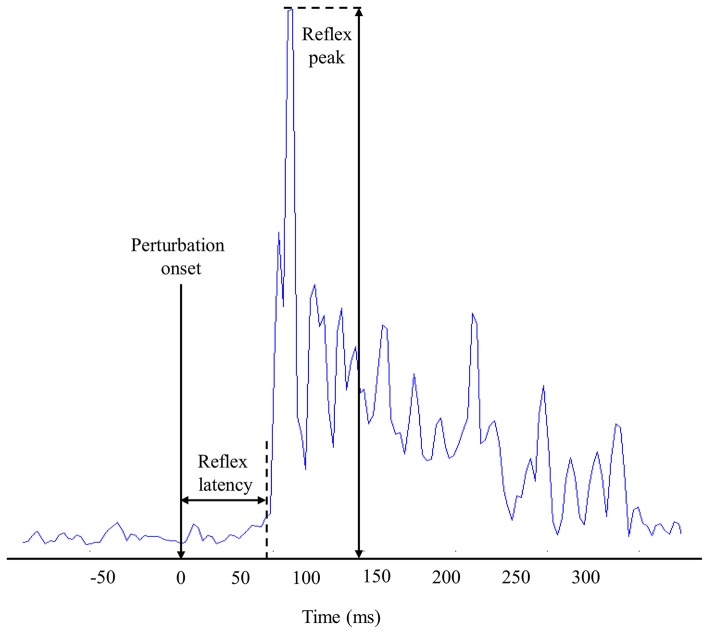
**Representation of muscle reflex variables extracted from one high-density electromyography (EMG) electrode during one perturbation trial**.

Reflex latency of erector spinae muscles was defined as the time delay from the perturbation onset to the EMG reflex onset. To calculate the reflex onset, EMG signals were Butterworth filtered (sixth-order, 50 Hz cut-off frequency) and assessed using a sliding window of 25-ms (Larivière et al., 2010). Muscle activity onset was then determined when the EMG signals exceeded three SD (Hodges and Bui, 1996) above the mean baseline EMG amplitude, which was calculated from a 1-s window before the perturbation onset (Figure 2). Reflex latency was also identified by a visual inspection of the EMG recordings by the same investigator. The reflex onset was defined as the beginning of the first peak EMG post perturbation that exceeded approximately two times the mean baseline activity. Due to the high number of electrodes, visual detection technique was only applied on four electrodes by trials. Mean of these four reflex latencies were used during subsequent statistical analyses.

#### Reflex Peak

The reflex peak corresponded to the highest RMS value following perturbation onset. Reflex peak had to be present in a 300-ms window following the perturbation onset to be considered a reflex response (Figure 2).

#### Area of Reflex Activity Spatial Distribution

**Figure 3:**
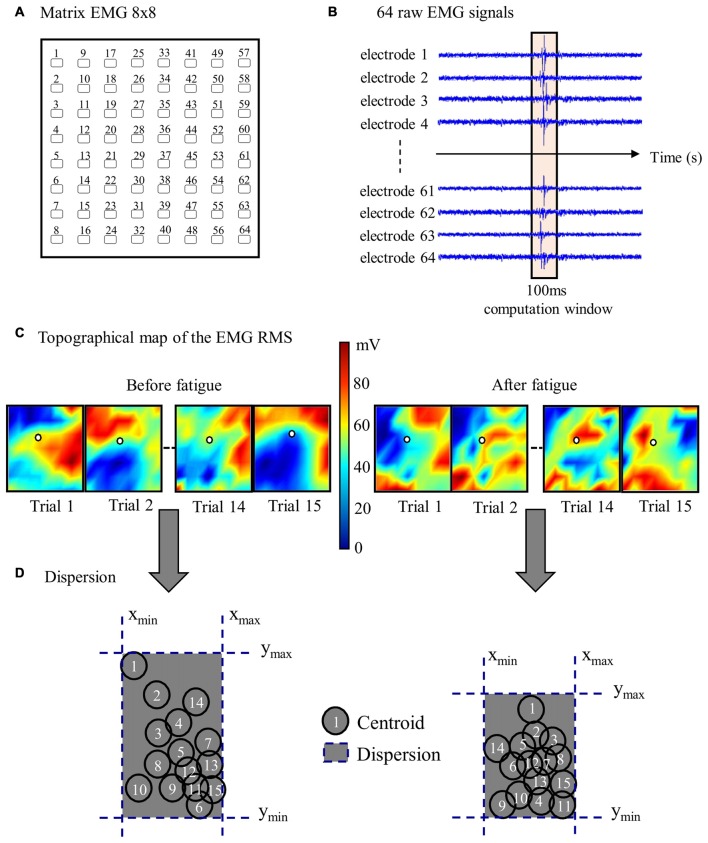
**Stages of high-density EMG data analyses. (A)** Representation of one 64-electrode matrix used in the recording of erector spinae muscle activity. **(B)** Myoelectric signals from 64 electrodes of one matrix in a random healthy participant during one perturbation trial. **(C)** Centroid migration from topographical representation of root mean square (RMS) reflex values computed within 100 ms windows at each trial. Note the difference between muscle activity recruitment pattern (color variation) between pre- and post-fatigue. **(D)** Dispersion representation from the 15 centroid position before and after the fatigue protocol.

To characterize reflex activity, spatial distribution of the dispersion variable representing the muscle activity range of displacement (centroid) was extracted from the bipolar EMG signals. As described in our previous study (Abboud et al., 2014), the centroid was defined as the mean RMS of all 64 electrodes of each high-density EMG. Specifically, the centroid value from each EMG signal was obtained by calculating the mean of RMS value from a window of 100-ms, divided equally (50-ms) on either side of the reflex peak. This operation was repeated through the 15 sudden perturbation trials pre- and post-fatigue protocol to produce dispersion values (see Figure 3 for more details). The dispersion of erector spinae muscles has been shown to be highly reliable (Abboud et al., 2015).

#### Abdominal Reflex Activity

Since reflex activity in the rectus abdominis and external obliquus rarely occurred after the perturbation, mean RMS values of the abdominal muscles were computed based on the same window of 100-ms used for erector spinae signal analyses. Since no difference was identified between left and right sides, mean values of left and right rectus abdominis as well as mean values of left and right external obliquus were used for the analyses.

#### Trunk Kinematic

Trunk kinematics were analyzed using the two adjacent LED to create a vector. Lumbar spine motion was obtained by calculating the trunk flexion angle between the T11-L1 vector and a horizontal vector relative to the ground. The angle values corresponded to the range of motion between the starting position before the perturbation, and the maximal trunk flexion following perturbation onset. From the trunk flexion angle, peak velocity and time to peak velocity were calculated. The third kinematic LED was used to determine the exact moment when the perturbation started.

### Statistical Analysis

Normality of distribution for every dependent variable was assessed using the Kolmogorov—Smirnov test, in addition to visual inspection of the data. Student *t*-tests for dependent samples were used to compare EMG amplitude RMS between MVC pre- and post-fatigue. Student *t*-tests for dependent samples were also used to compare muscle activity spatial distribution before and following the fatigue task. The first trial pre-fatigue was also compared to the first trial post-fatigue for each dependent variables using *t*-tests for dependent samples. A mixed model two-way repeated measure ANOVA was conducted to assess: (1) the adaptation effect across trials; (2) the fatigue effect; and (3) the interaction effect (fatigue × adaptation) for each dependent variable (baseline activity, reflex latency, reflex peak, abdominal reflex activity and trunk kinematic). For the variables: baseline, reflex latency, reflex peak and kinematic variables, the means of the first and last five perturbation trials before and after fatigue were considered for the two-way repeated measures ANOVA. When necessary, the Tukey *post hoc* test was performed for pair-wise comparisons. Repeated measures ANOVA followed by quadratic polynomial contrast trend analyses were also conducted for reflex peak values to assess adaptation across perturbation trials before and after muscle fatigue. The reliability of the reflex latency values were estimated by the intraclass correlation (ICC, type 3,1). ICC_3,1_ evaluated inter-rater reliability, using the Matlab software (MathWorks; Natick, MA, USA) representing one rater and visual detection the other one. The standard error of measurement (SEM) was also assessed using the formula SEM = SD * √1 − ICC. For all statistical analyses, *p* < 0.05 was considered to be statistically significant.

## Results

From the 25 original participants, one participant was excluded from all analyses due to the impossibility of identifying the beginning of the perturbation. Moreover, 2% of all perturbation trials from high-density sEMG were not considered for the analyses due to the absence of a reflex response. As for abdominal muscle reflex activity analyses, three participants were excluded due to the poor quality of EMG signals.

### Presence of Low Back Muscle Fatigue

The mean endurance time of the fatigue protocol was 125 s (*SD* = 39.4). During the fatigue protocol, a negative slope of MDF values was observed. MDF slope values were −0.184 (*SD* = 0.09) on the right side and −0.165 (*SD* = 0.09) on the left side of the erector spinae. As expected, a significant reduction of EMG amplitude RMS was found between MVC pre- and post-fatigue on the right side of the erector spinae (*p* ≤ 0.001) and on the left side (*p* ≤ 0.001). Moreover, reductions of EMG amplitude RMS of 28% and 24% on the right and left side, respectively, were found. The perceived effort mean score was 17.6 (*SD* = 1.7), which corresponds to the “very hard” level of perceived effort on the Borg scale. Such level is described as the highest level of activity you can sustain (Borg, 1982).

### First Perturbation Trial

In most of cases, the first perturbation trial response pre- and post-fatigue was not affected by the presence of muscle fatigue. Dependent *t*-tests revealed no significant difference between the first perturbation trial before fatigue and the first perturbation trial after fatigue on both sides of the erector spinae muscles for the baseline activity (right side, *p* = 0.55; left side, *p* = 0.14), the reflex latency (right side, *p* = 0.84; left side, *p* = 0.49), the reflex peak (right side, *p* = 0.91; left side, *p* = 0.29), as well as for the rectus abdominis (*p* = 0.41) and external obliquus (*p* = 0.05) reflex amplitudes. As for the kinematic variables, dependent *t*-tests revealed no significant difference between the first perturbation trial before fatigue and the first perturbation trial after fatigue for the trunk flexion angle (*p* = 0.78) and the peak velocity (*p* = 0.46), while a significant decrease between the first perturbation trial before fatigue and the first perturbation trial after fatigue was found for time to peak velocity (*p* = 0.007).

### Trunk Kinematic

Most of the trunk kinematic variables did not change with the presence of muscle fatigue and did not adapt over perturbation trials. Only the time to peak velocity were found to be altered. The mixed model repeated measure ANOVA revealed no significant adaptation effect across trials (*F*_(1,23)_ = 1.06, *p* = 0.31) nor a main effect of fatigue (*F*_(1,23)_ = 0.22, *p* = 0.64) for the trunk flexion angle. As for peak velocity, the analyses showed no main effect of fatigue (*F*_(1,23)_ = 0.72, *p* = 0.41) and no adaptation effect (*F*_(1,23)_ = 3.56, *p* = 0.07). Results from the mixed model repeated measure ANOVA also showed a main effect of fatigue for time to peak velocity, with a longer time to peak velocity before the fatigue task (*F*_(1,23)_ = 27.25, *p* ≤ 0.001). Additionally, a significant reduction of time to peak velocity was found, representing a main effect of adaptation across trials (*F*_(1,23)_ = 7.01, *p* = 0.01; see Table 1 for mean and SD values).

**Table 1:** **Mean values SD of the first and last five perturbation trials before and after the fatigue protocol (maximal voluntary contraction, MVC; L, left side of the erector spinae; R, right side of the erector spinae)**.

			First five trials mean	Last five trials mean	p*	
					Fatigue	Adaptation
Flexion angle (°)		Pre-fatigue	6.2 (4.0)	5.9 (4.1)	*p* = 0.64	*p* = 0.31
		Post-fatigue	6.0 (3.9)	5.8 (3.4)		
Peak velocity (°/s)		Pre-fatigue	21.3 (10.8)	19.5 (9.4)	*p* = 0.41	*p* = 0.07
		Post-fatigue	21.8 (10.8)	20.5 (7.1)		
Time to peak velocity (ms)		Pre-fatigue	238 (86)	217 (86)	*p* ≤ 0.001	*p* = 0.01
		Post-fatigue	205 (67)	187 (79)		
Baseline (% MVC)	L	Pre-fatigue	8.2 (4.1)	7.6 (3.9)	*p* = 0.03	*p* = 0.02
		Post-fatigue	9.6 (5.1)	9.1 (4.6)		
	R	Pre-fatigue	9.9 (5.2)	9.6 (5.6)	*p* = 0.18	*p* = 0.57
		Post-fatigue	10.7 (6.1)	10.6 (5.7)		
Reflex latency (ms)	L	Pre-fatigue	94.3 (31.9)	89.3 (32.5)	*p* = 0.19	*p* = 0.08
		Post-fatigue	102.1 (39.2)	96.1 (42.4)		
	R	Pre-fatigue	93.8 (29.5)	98.2 (44.8)	*p* = 0.75	*p* = 0.25
		Post-fatigue	95.9 (39.1)	97.9 (36.7)		
Reflex peak (% MVC)	L	Pre-fatigue	60.1 (26.7)	49.5 (23.9)	*p* = 0.38	*p* ≤ 0.001
		Post-fatigue	60.4 (26.9)	54.4 (24.4)		
	R	Pre-fatigue	66.7 (25.6)	52.5 (17.6)	*p* = 0.02	*p* ≤ 0.001	
		Post-fatigue	70.2 (27.9)	65.4 (25.1)		

*p based on the repeated measures ANOVA.

### Baseline Activity

Small changes were observed in baseline activity after the muscle fatigue protocol as well as over the perturbation trials. The analyses yielded a main effect of fatigue for baseline activity with a higher baseline value after the fatigue task on the left side (*F*_(1,23)_ = 5.18, *p* = 0.03), but not on the right side (*F*_(1,23)_ = 1.90, *p* = 0.18). Similarly, a significant main effect of adaptation, represented by a reduction of baseline activity through the perturbation trials, was observed on the left side (*F*_(1,23)_ = 6.63, *p* = 0.02), but not on the right side (*F*_(1,23)_ = 0.33, *p* = 0.57; see Table 1 for mean and SD values).

### Reflex Latency

Erector spinae reflex latency remained unchanged with or without muscle fatigue and over the perturbation trials. The mixed model repeated measure ANOVA showed no significant main effect of fatigue on both sides of the erector spinae (for the right side (*F*_(1,23)_ = 0.11, *p* = 0.75); for the left side (*F*_(1,23)_ = 1.80, *p* = 0.19). No significant adaptation effect was observed on either sides (for the right side (*F*_(1,23)_ = 1.41, *p* = 0.25); for the left side (*F*_(1,23)_ = 3.34, *p* = 0.08); see Table 1 for mean and SD values). The ICC obtained for reflex latency values was moderate (ICC_3,1_ = 0.63, 95% CI = 0.31–082) and the SEM was small (SEM = 0.016).

### Reflex Peak

**Figure 4:**
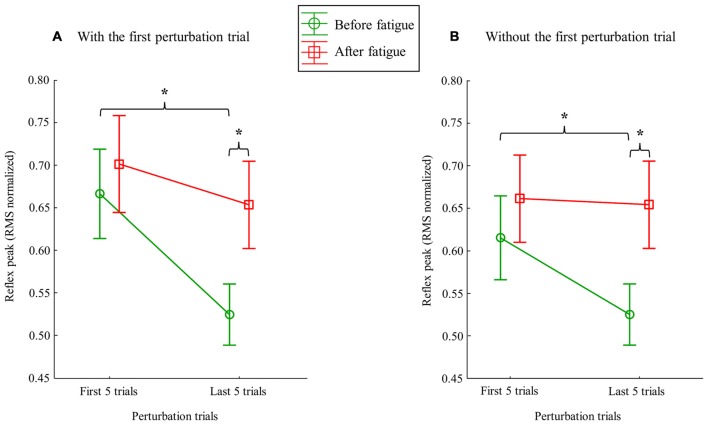
**Mean RMS peak results on the right side with (A)** and without **(B)** the first perturbation trial on the right side. Error bars indicate standard errors. Significant *post hoc* results are illustrated by *p ≤ 0.001.

Following the muscle fatigue task, erector spinae reflex peak value was increased, while adaptations over perturbation trials were altered. Results from the mixed model repeated measure ANOVA showed a main effect of fatigue for reflex peak with a higher peak value after the fatigue task on the right side (*F*_(1,23)_ = 6.47, *p* = 0.02), but not on the left side (*F*_(1,23)_ = 0.80, *p* = 0.38). Moreover, a significant main effect of adaptation, represented by a reduction of reflex peak amplitude through the perturbation trials, was observed on the right side (*F*_(1,23)_ = 19.55, *p* ≤ 0.001), and on the left side (*F*_(1,23)_ = 19.70, *p* ≤ 0.001). The analyses also showed a significant fatigue × adaptation interaction effect on the right side (*F*_(1,23)_ = 7.68, *p* = 0.011); a similar tendency, although not significant, was observed on the left side (*F*_(1,23)_ = 3.16, *p* = 0.089; see Table 1 for mean and SD values). As illustrated in Figure 4, *post hoc* analyses revealed higher reflex peak values in the first perturbation trials vs. the last ones in the condition pre-fatigue, but not under the influence of muscle fatigue (*p* ≤ 0.001). Moreover, a significant higher peak reflex value was found in the last perturbation trials after vs. before fatigue protocol (*p* ≤ 0.001).

**Figure 5:**
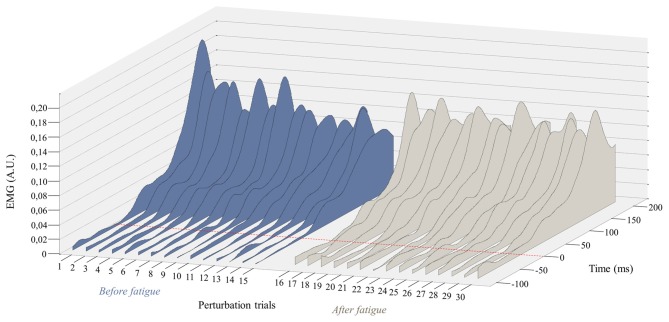
**Representation of the mean EMG activity traces for the right erector spinae before (perturbation trials 1–15) and after (perturbation trials 16–30) the fatigue task.** The red dotted line represents the perturbation onset. (A.U. Arbitrary Unit).

Since the first trial reaction is known to have a higher impact on postural balance (Allum et al., 2011), all these analyses were performed a second time without taking into account the first trial before and after the fatigue protocol. This procedure was conducted to explore whether or not the reflex peak attenuation observed over perturbation trials was only due to the first trial. Once again, results from the repeated measure ANOVA showed a main effect of fatigue for reflex peak with a higher peak value after the fatigue task on the right side (*F*_(1,23)_ = 8.35, *p* = 0.008; Figure 5), but not on the left side (*F*_(1,23)_ = 2.02, *p* = 0.17). A significant main effect of adaptation was also observed on the right side (*F*_(1,23)_ = 6.99, *p* = 0.015), and on the left side (*F*_(1,23)_ = 9.66, *p* = 0.005). The analyses also showed a significant fatigue × adaptation interaction effect on the right side (*F*_(1,23)_ = 12.09, *p* = 0.002), but not on the left side (*F*_(1,23)_ = 0.96, *p* = 0.33). As illustrated in Figure 4, *post hoc* analyses showed higher reflex peak values in the first perturbation trials vs. the last ones in the condition pre-fatigue, but not under the influence of muscle fatigue (*p* ≤ 0.001). *Post hoc* analyses also revealed that a significant higher peak reflex value was found in the last perturbation trials after the fatigue protocol vs. the last trials performed before muscle fatigue (*p* ≤ 0.001).

Polynomial quadratic trend analyses yielded a significant adaptation (decreasing response) before the fatigue protocol on both sides (for the right side *p* = 0.01, contrast estimate of 0.99; for the left side *p* = 0.007, contrast estimate of 1.02), but not under the influence of muscle fatigue (for the right side *p* = 0.45, contrast estimate of 0.35; for the left side *p* = 0.26, contrast estimate of 0.46).

### Reflex Spatial Distribution

A higher reflex spatial distribution was observed before the fatigue protocol. Dependent *t*-tests revealed a significant difference between the protocol before fatigue (*M* = 1.13, *SD* = 0.25) and after (*M* = 0.95, *SD* = 0.29) for the dispersion variable on the left side of the erector spinae (*p* = 0.02). A higher dispersion value, yet not significant (*p* = 0.08), was found on the right side before the fatigue (*M* = 1.16, *SD* = 0.34) when compared to trials performed following fatigue (mean = 1.03, *SD* = 0.30).

Dependent *t*-tests were repeated a second time without taking into account the first trial before and after the fatigue protocol. Results from the left side also revealed a significant higher value before the muscle fatigue (*M* = 1.10, *SD* = 0.22) compared to the trials following muscle fatigue (*M* = 0.93, *SD* = 0.29; *p* = 0.02). Once again, a higher dispersion value, yet not significant (*p* = 0.07), was found on the right side before the fatigue (*M* = 1.12, *SD* = 0.35) compared to the trials following muscle fatigue (*M* = 0.99, *SD* = 0.29).

**Figure 6:**
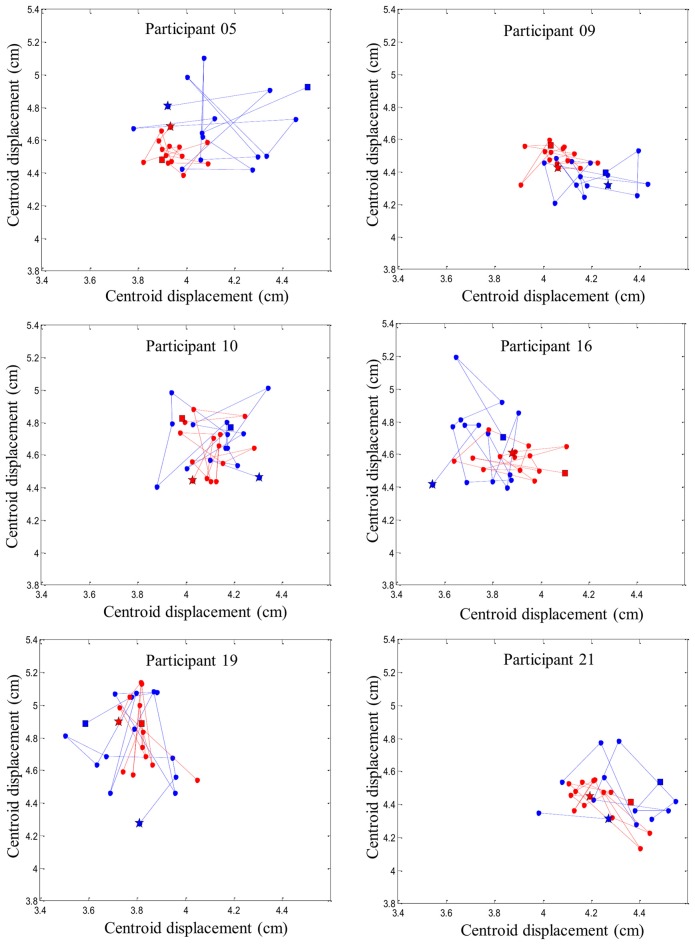
**Representation of six random participants’ centroid displacement between perturbation trials on the right erector spinae muscles.** Blue line represents centroid displacement before the fatigue task. Red line represents centroid displacement after the fatigue task. Stars represent the first trials and squares represent the last trials of each condition (pre- and post-fatigue).

Figure 6 provides an illustration of the complex and variable muscle activity distribution pattern during the perturbation trials before and after the fatigue task. Results showed a smaller centroid migration through the perturbation trials after the fatiguing task.

### Abdominal Reflex Activity

Results from the repeated measures ANOVA showed a main effect of fatigue for abdominal reflex activity with a decrease of reflex activity after the fatigue task for the rectus abdominis (*F*_(1,20)_ = 8.82, *p* = 0.008) and the external obliquus (*F*_(1,20)_ = 9.50, *p* = 0.006). Moreover, a significant main effect of adaptation, showing a reduction of abdominal reflex activity, was found only for the rectus abdominis (*F*_(1,20)_ = 8.01, *p* = 0.01), but not for the external obliquus (*F*_(1,20)_ = 0.79, *p* = 0.39).

## Discussion

Understanding the neuromuscular responses to unexpected loading of the trunk is highly relevant in view of everyday life and to the investigation of spinal stability and movement control. The present study assessed how erector spinae muscle adapts after a fatigue task fatigue following a series of repeated sudden external perturbations. Using high-density sEMG, this study is the first one showing variability in lower back muscle activity recruitment pattern strategies with a condition perturbing spinal stability. Moreover, this neuromuscular adaptation was altered following back muscle fatigue.

### Methodological Considerations

Some fatigue recovery may have occurred over the 15 perturbation trials following the fatigue protocol. Several measures were taken to limit attenuation of muscle fatigue effects. The transition between the fatigue protocol and the second series of perturbation was made as quickly as possible. The time needed between the end of the fatigue protocol and the acquisition of the data ranged from 2 min to 4 min, while it took less than 8 min to conduct the 15 perturbation trials. A study demonstrated that recovery from back muscle fatigue occurs after approximately 10–15 min of rest (Larivière et al., 2003). However, in this study, participants had to perform a 30 s trunk extension at 75% of their MVC. In the present study, there was no time-limit for the fatigue task and participants were encouraged to maintain position until total exhaustion was reached.

The different acquisition frequencies used in EMG (2048 Hz) and kinematics (100 Hz) system can be considered as a methodological limitation of the study. The difference between acquisition frequencies could lead, under a worst case scenario, to a 10-ms margin of error, when identifying the onset of perturbations. However, the perturbation onset was only used to compute the reflex latencies and consequently did not affect other EMG variables.

### Muscle Fatigue Effect

During the experiment, muscle fatigue manifestation in the erector spinae muscles was confirmed by a marked decreased in the MDF slope (De Luca, 1984; Mannion and Dolan, 1994). Moreover, participants perceived the fatigue task as a very hard exertion (Borg, 1982). Finally, a decrease in erector spinae EMG amplitude during post-fatigue MVC is also considered as a valid indicator of muscle fatigue (Enoka and Duchateau, 2008). These observations taken together suggest that the participant were in a fatigue state following the fatigue task. Regarding the impact of muscle fatigue on kinematic variables, results showed no difference between pre- and post-fatigue for trunk flexion angle following the perturbation. This observation is in accordance with previous studies that have also demonstrated that trunk flexion angle is not affected by acute back muscle fatigue following an unexpected perturbation (Granata et al., 2004; Mawston et al., 2007). Moreover, participant peak velocities remained unchanged with the presence of muscle fatigue. On the other hand, the present study is the first one showing that in the presence of muscle fatigue, participants decreased their time to peak velocity in response to the perturbation. These results suggest that fatigue did induce some changes in the neuromuscular control of postural balance, but the sensorimotor system remained partly efficient when the low back region was fatigued. This strategy could be explained by the trunk muscle system’s redundancy which offers various adaptation possibilities to achieve a similar goal (Latash and Anson, 2006). Even if the trunk flexion angle were similar with or without fatigue, there were probably other neuromuscular strategies that prevented the effect of fatigue, such as variability in muscle activity recruitment pattern (see “New Insights into Motor Adaptation to Spinal Instability” Section for further explanation).

Using high-density sEMG, this study is the first one exploring EMG reflex variables with muscle fatigue. In the current study, baseline activity seemed to show a slight trend towards higher muscle activation after the fatigue protocol (only significant on one side of the erector spinae). Current evidence have not find a common understanding on baseline activity prior a perturbation under the influence of muscle fatigue (Granata et al., 2001, 2004; Herrmann et al., 2006; Mawston et al., 2007; Grondin and Potvin, 2009; Dupeyron et al., 2010). Baseline activity is directly linked to EMG amplitude signals, which corresponds to the number of active motor units. Muscle fatigue is characterized by an increase of active motor units (De Luca, 1997), which is usually reflected by an increase in EMG amplitude signals in submaximal muscle contractions. A recent review has shown that muscle fatigue induced by a submaximal isometric contraction is associated with variable responses in motor units firing rates according to the intensity of the fatiguing task (Taylor et al., 2016). For example, motor unit behavior during submaximal isometric contraction at moderate intensity (50% MVC), which correspond to the Sorensen test (Demoulin et al., 2006), is first associated with a decrease in firing rate followed by an increased motor units recruitment (Heckman and Enoka, 2012). Since others studies have used different fatigue protocol, with effort intensity varying from approximately 20–60% of the MVC (Granata et al., 2001; Herrmann et al., 2006; Dupeyron et al., 2010), a variation in motor unit behavior could partly explain discrepancies across studies. In the current study, two methods were used to determine the exact time between the onset of the perturbation and the reflex response. Despite a moderate reliability between those detection methods (ICC 0.63), reflex latency values (≈95 ms) are similar to those observed in the studies (ranging from ≈60 ms to 125 ms) measuring the impact of muscle fatigue on reflex latency (Chow et al., 2004; Granata et al., 2004; Herrmann et al., 2006; Dupeyron et al., 2010; Sánchez-Zuriaga et al., 2010). Furthermore, no change was observed under the influence of muscle fatigue in the present study. This observation is consistent with most of the studies (Granata et al., 2004; Herrmann et al., 2006; Dupeyron et al., 2010; Sánchez-Zuriaga et al., 2010). In the literature, reflex amplitude of low back muscles was found not to be affected by muscle fatigue in several studies (Granata et al., 2004; Grondin and Potvin, 2009; Sánchez-Zuriaga et al., 2010), whereas two studies found a higher back reflex amplitude following a fatigue protocol (Herrmann et al., 2006; Dupeyron et al., 2010). In the present study, an increase in reflex peak values was observed after the fatiguing task. An interesting assumption to explain the discrepancy in reflex peak results could be the presence of an association between baseline activity responses, reflex peak and muscle fatigue. Indeed, studies who have reported an increase in baseline activity are the same that did not observe a change in reflex peak amplitude after muscle fatigue (Granata et al., 2004; Grondin and Potvin, 2009), and vice versa (Herrmann et al., 2006; Dupeyron et al., 2010). It could be hypothesized that increased muscle pre-activation is sufficient to counteract the fatigue effect in response to an external perturbation. On the other hand, with negligible change in pre-activation level, as observed in our study, neuromuscular adaptations are reflected in the variation of reflex peak amplitude.

In parallel to the observation of an increased reflex activity amplitude in the back muscle following fatigue, a decreased abdominal reflex activity was identified. This observation suggests that an increase in erector spinae activity would be sufficient to increase spinal stability in order to compensate for acute back fatigue effect (Cholewicki et al., 2000; Andersen et al., 2004). However, these results should be interpreted with caution since abdominal muscle EMG reflex amplitude remained higher than 50% of their activity during the MVC.

### Trial-to-Trial Adaptation

In the current study, participants were submitted to a series of the exact same unexpected perturbation of the trunk. Kinematic variables including trunk flexion angles and velocity peaks remained constant through the perturbation trials. However, a decrease in time to peak velocity was observed across trials of the same unexpected perturbation. This suggest that participants adapted to the unexpected perturbation by taking less time to stop their trunk movement. A group of authors have also shown, across 10 trunk perturbation trials, a progressive reduction in the time interval between forward trunk movement initiation and complete cessation of trunk movement (Skotte et al., 2004). These observations suggest that, when first facing an unexpected perturbation, the sensorimotor systems allows irrelevant components of a motor task to fluctuate. According to the minimal intervention principle, the irrelevant aspects from the resulting behavior should be left uncorrected in order to maximize motor performance (Todorov and Jordan, 2002). Based on this principle and the findings of this study, one could argue that trunk movements triggered by the perturbations (6° on average) were not sufficient, and consequently no adaptation of the trunk flexion angle was needed to optimize spinal stabilization. In such context, trunk flexion angle and peak velocity would be considered irrelevant aspect of spinal stability while time to peak velocity would have more significant consequences on stability and potential tissue damage.

Across perturbation trials, a small attenuation of baseline activity was observed, while no adaptation was found for the reflex latency. The absence of reflex latency trial-to-trial adaptation was also observed in a previous study (Skotte et al., 2004). As for reflex peak amplitude, a clear reduction of amplitude values through the repetition of the unexpected trunk perturbation was found in the present study. It is known that the first trial reaction to an unexpected perturbation has a higher impact on postural balance in standing or seated positions (Allum et al., 2011). Results from the present study have shown that even without considering the first trial response, an attenuation of the reflex response still occurred. However, it is important to note that as we get closer to the last perturbation trial, the attenuation lessens. Similar observations were found following unexpected tilts of a surface platform, with authors showing a rapid adaptation of EMG amplitude between the first two trials, whereas adaptation was more gradual over the next trials (Oude Nijhuis et al., 2010). Again, based on this motor behavior, it could be suggested that, in the presence of an unknown movement or perturbation, the optimal strategy would be to first adopt a broader but less specific motor response (Todorov and Jordan, 2002). Following repetitions of the same unexpected perturbation, the trunk muscle system’s redundancy could offer corrections by using the appropriate number of degrees of freedom (Latash and Anson, 2006). Such adaptations would also explain the progressive time to peak velocity reduction across perturbation trials.

Interestingly, this progressive decrease continued throughout perturbation trials following the fatigue task. This suggests that even when muscle fatigue is present, participants continued their adaptation to perturbations by taking less time to stabilize their trunk. Since trunk flexion angle and velocity peak remained unchanged, it suggests that participants were able to stabilize trunk movement using alternative neuromuscular strategies. Indeed, following muscle fatigue, the trial-to-trial adaptation of the EMG reflex peak appeared to be limited. To our knowledge, the present study is the first one showing that adaptation of the reflex peak across perturbation trials is altered following muscle fatigue. The amplitude of the reflex peak remained almost constantly at the same level from the first to the last external perturbation. It could be hypothesized that muscle fatigue limited the possibility of using across-trial redundancy to adapt and optimize spinal stabilization using retroactive control. Moreover, since postural balance, expressed as a trunk angle, also remained constant throughout the perturbation trials with or without muscle fatigue, it could be suggested that alterations of the EMG reflex adaptation are an attempt to preserve a constant postural balance despite the presence of muscle fatigue.

As discussed in the methodological considerations section, some recovery may have been present throughout the last perturbation trials. In a previous study, the effect of recovery from muscle fatigue on adaptation to external perturbations was explored (Takahashi et al., 2006). Results showed that recovery affects recall of the internal model. The authors suggested that participants overestimated the muscle activity required to counteract the perturbation because their muscle force-generation capacity recovered during rest. Although fatigue and recovery effects cannot be teased out, results from the present study show that following a fatigue protocol, a modification of EMG reflex peak and trunk kinematics (time to peak velocity) occurred whereas trial-to-trial adaptation in EMG reflex peak was found to be limited following a protocol of fatigue.

Finally, the adaptation throughout the first perturbation trial following muscle fatigue was almost inexistent. Results from the present study showed that only time to peak velocity was affected when participants experienced, for the first time, an unexpected perturbation following a fatigue task. Similarly to the adaptation phenomenon observed across perturbation trials, participants took less time to stabilize their trunk following the fatigue protocol. These findings suggest that in most cases, the neuromuscular component of spinal stability is not significantly challenged in the presence of muscle fatigue, regardless of the number of exposure to a specific trunk perturbation.

### New Insights into Motor Adaptation to Spinal Instability

Using high-density sEMG, dispersion of muscle activity, representing the area of reflex activity spatial distribution, was used to better understand trial-to-trial adaptation with and without muscle fatigue. A higher variability in muscle activity spatial distribution was observed before muscle fatigue was induced, while under the influence of fatigue, a reduction of the centroid migration was found. Changes in muscle activity distribution to different regions of the lumbar erector spinae could be associated with changes in variation in the control of motor units within this muscle. As mentioned earlier, adaptation of muscle reflex activity occurred through the repetition of similar sudden external perturbations. This observation suggests that the trial-to-trial adaptation was associated with higher levels of muscle activity spatial distribution. Conversely, such adaptation was not present when erector spinae muscles were in a state of fatigue. This suggests that changing spatial distribution of EMG activity, consistent with increased motor variability (Latash, 1998), may help face a series of unexpected perturbation. On the other hand, the reduced muscle activity spatial distribution observed in presence of muscle fatigue could be explained by the initiation of protective and restrictive neuromuscular strategies. This study suggests that under the influence of fatigue, the nervous system chooses to adopt a more stable muscle activity distribution when spinal stability is challenged. This observation are complementary with the work of Missenard et al., which showed that muscle fatigue increases the variance of motor commands during voluntary movements (Missenard et al., 2008, 2009). Motor variability can be measured using multiple parameters, such as muscle activity, kinetic or kinematic components of the movement pattern, or external force developed (Srinivasan and Mathiassen, 2012). In the present study, a more stable pattern of muscle activity distribution following fatigue, can be explained by the decreased number of motor unit available to generate muscle responses during perturbations. This reduced number of available motor unit may lead to more stable spatial distribution as illustrated by the reduced centroid migration in the presence of muscle fatigue (Figure 6). Moreover, muscle fatigue by reducing the number of available motor units may increase the variability in movement patterns (Missenard et al., 2008, 2009).

It is also important to note that the migration of muscle activity was highly variable between participants before and after the fatigue task. While spatial muscle activity distribution has been shown to shift laterally and caudally during a fatigue task in the low back region (Abboud et al., 2014), no distinctive muscle activity migration pattern was identified following a repeated unexpected perturbation. A recent study has provided similar results when a group of healthy participants were injected with a saline solution to induce acute low back pain (Hodges et al., 2013). After the injection, none of the participants activated their trunk muscles in the same manner in response to an external trunk perturbation (Hodges et al., 2013). These results reflect the complexity of the redundant trunk system, which offers various motor possibilities to achieve a similar goal. This raises the question whether these adaptations are maintained across various perturbation tasks or if the nervous system triggers individual similar adaptations for each person. Further research will be needed to verify if the alteration of trial-to-trial response is also present in a different external environment trunk perturbation such as chronic low back pain or spinal tissue creep.

In conclusion, the results of the present study suggest that participants adapt differently under the influence of muscle fatigue when they experience an unknown perturbation. While the EMG reflex amplitude remains constant over perturbation trials after the fatigue task, participants continue to habituate their trunk movements. Moreover, this study suggests that the nervous system chooses to adopt a more restrictive muscle activity recruitment pattern, when the unknown perturbation is repeated after a muscle fatigue task. Since this study is the first one describing such motor adaptations, it seems reasonable to propose that following muscle fatigue, the motor system can still generate proper stabilizing responses to spinal perturbations using alternative strategies. These strategies, however, may have detrimental long-term consequences that should also be considered in the context of spine rehabilitation.

## Author Contributions

JA, FN, AL, CD and MD: substantial contributions to the conception or design of the work, or acquisition, analysis, or interpretation of data for the work; drafting the work and revising it critically for important intellectual content; final approval of the version to be published; agreement to be accountable for all aspects of the work in ensuring that questions related to the accuracy or integrity of any part of the work are appropriately investigated and resolved.

## Funding

This study was funded through the Natural Sciences and Engineering Research Council of Canada in the form of a scholarship.

## Conflict of Interest Statement

The authors declare that the research was conducted in the absence of any commercial or financial relationships that could be construed as a potential conflict of interest.
